# Kinetic Analysis of Substrate Utilization by Native and TNAP-, NPP1-, or PHOSPHO1-Deficient Matrix Vesicles

**DOI:** 10.1359/jbmr.091023

**Published:** 2009-10-17

**Authors:** Pietro Ciancaglini, Manisha C Yadav, Ana Maria Sper Simão, Sonoko Narisawa, João Martins Pizauro, Colin Farquharson, Marc F Hoylaerts, José Luis Millán

**Affiliations:** 1 Sanford Children's Health Research Center, Sanford-Burnham Medical Research InstituteLa Jolla, CA, USA; 2 Departmento Química, FFCLRP-USPRibeirão Preto, São Paulo, Brazil; 3 Departmento TecnologiaFCAVJ-UNESP, Jaboticabal, São Paulo, Brazil; 4 Bone Biology Group, Roslin Institute, University of EdinburghScotland, UK; 5 Center for Molecular and Vascular Biology, University of LeuvenLeuven, Belgium

**Keywords:** biomineralization, knockout mice, calcification, pyrophosphatases, atpases

## Abstract

During the process of endochondral bone formation, chondrocytes and osteoblasts mineralize their extracellular matrix by promoting the formation of hydroxyapatite seed crystals in the sheltered interior of membrane-limited matrix vesicles (MVs). Here, we have studied phosphosubstrate catalysis by osteoblast-derived MVs at physiologic pH, analyzing the hydrolysis of ATP, ADP, and PP_i_ by isolated wild-type (WT) as well as TNAP-, NPP1- and PHOSPHO1-deficient MVs. Comparison of the catalytic efficiencies identified ATP as the main substrate hydrolyzed by WT MVs. The lack of TNAP had the most pronounced effect on the hydrolysis of all physiologic substrates. The lack of PHOSPHO1 affected ATP hydrolysis via a secondary reduction in the levels of TNAP in PHOSPHO1-deficient MVs. The lack of NPP1 did not significantly affect the kinetic parameters of hydrolysis when compared with WT MVs for any of the substrates. We conclude that TNAP is the enzyme that hydrolyzes both ATP and PP_i_ in the MV compartment. NPP1 does not have a major role in PP_i_ generation from ATP at the level of MVs, in contrast to its accepted role on the surface of the osteoblasts and chondrocytes, but rather acts as a phosphatase in the absence of TNAP. © 2010 American Society for Bone and Mineral Research.

## Introduction

Mineralization of cartilage and bone occurs by a series of physicochemical and biochemical processes that together facilitate the deposition of hydroxyapatite (HA) in specific areas of the extracellular matrix (ECM). Experimental evidence has pointed to the presence of HA crystals along collagen fibrils in the ECM,(1) as well as within the lumen of chondroblast- and osteoblast-derived matrix vesicles (MVs).(2) Investigators in the bone mineralization field generally are divided in supporting the collagen- versus the MV-mediated mechanism of mineralization. We see no incompatibility between these mechanisms. Our working model is that bone mineralization is first initiated within the lumen of MVs, and in a second step, HA crystals grow beyond the confines of the MVs and become exposed to the extracellular milieu, where they continue to propagate along collagen fibrils. This process is orchestrated by the balanced action of promoters and inhibitors of calcification.(3,4)

A primary inhibitor of ECM mineralization is extracellular inorganic pyrophosphate (PP_i_),(5) produced ectoplasmically by the enzymatic action of nucleotide pyrophosphatase/phosphodiesterase-1 (NPP1) that catabolizes extracellular ATP to produce PP_i_ and AMP.(6) Intracellular PP_i_ is also transported to the extracellular mileu by the channeling function of the ankylosis protein (ANK).(7) Tissue-nonspecific alkaline phosphatase (TNAP)(8) plays the crucial role of restricting the concentration of extracellular PP_i_ to maintain a P_i_/PP_i_ ratio permissive for normal bone mineralization.(9–11) Mice deficient in NPP1 (*Enpp1*^*−/−*^) or ANK (*ank/ank*) develop soft tissue calcification, including vascular calcification, resulting from the reduced production or transport of PP_i_,(10,12,13) whereas mice deficient in TNAP function (*Akp2*^*−/−*^) display rickets and osteomalacia owing to an arrest in the propagation of HA crystals outside the MVs caused by an increase in extracellular PP_i_ concentrations.(14–17) HA crystals are still present in TNAP-deficient MVs,(16) and it has been proposed that the soluble MV phosphatase PHOSPHO1, with specificity for phosphoethanolamine and phosphocholine,(18) might be involved in increasing the local intravesicular concentration of P_i_ to change the P_i_/PP_i_ ratio in favor of precipitation of HA seed crystals.(19) Finally, the coexpression of TNAP and type I collagen is necessary to cause mineralization of any ECM, indicating that propagation of HA crystals in the bone ECM is intimately dependent on the presence of type I collagen.(11)

While the substrate specificities for TNAP, NPP1, and PHOSPHO1 have been established using purified enzymes in vitro, here we have set out to examine substrate utilization by isolated osteoblast-derived MVs, where these and other enzymes are present together in a physiologic biologic compartment. Here we report the relative ability of WT MVs, as well as MVs deficient in TNAP, NPP1, or PHOSPHO1, to use the substrates ATP, ADP, and PP_i_ under physiologic conditions.

## Materials and Methods

### MV isolation

The production and characterization of the *Akp2*^*−/−*^(14) and *Enpp1*^*−/−*^(20) mice has been reported. The *Phospho1*^*−/−*^ mice were identified and selected from a screen of *Phospho1*-inactivating mutations of the *N*-ethyl-*N*-nitrosourea (ENU) mutagenesis sperm bank collection of Ingenium Pharmaceuticals AG (Martinsried, Germany). This strain carries a C>T mutation in codon 74 of exon 2 of the *Phospho1* gene that leads to a premature stop codon (R74X) and the complete absence of PHOSPHO1 protein (not shown).

MVs were isolated from primary mouse calvarial osteoblasts removed from 1- to 3-day-old WT, *Akp2*^*−/−*^, *Enpp1*^*−/−*^, and *Phospho1*^*−/−*^ pups by collagenase digestion. Osteoblasts were plated in 10 cm plates at a density of 0.75 × 10^6^ in α modified essential medium (α-MEM, Invitrogen, Carlsbad, CA, USA) containing 10% fetal bovine serum (FBS). On the next day, the medium was replaced with differentiation medium (α-MEM with 10% FBS and 50 µg/mL ascorbic acid). The cells were grown for 18 days, with a medium change every third day. The cell monolayer was washed with medium without FBS and digested in a collagenase digestion mixture containing 0.45% collagenase (Worthington, Lakewood, NJ, USA), 0.12 mol/L NaCl, 0.01 mol/L KCl, 1000 U/mL of penicillin, 1 mg/mL of streptomycin, and 0.05 mol/L Tris buffer (pH 7.6) at 37°C. Collagenase digestion was done at 37°C for 1.5 to 2 hours. The collagenase digest then was centrifuged at 3500 rpm for 10 minutes to harvest cells. The supernatant was subjected to a two-step differential ultracentrifugation for the isolation of MVs. The first step involves the centrifugation of collagenase digest at 19,500 rpm for 10 minutes to remove nuclei, mitochondria, lysosomes, and smaller cell fragments. The supernatant then was further centrifuged at 42,000 rpm for 45 minutes to obtain a highly pure MV pellet. The yield of MVs was estimated by measuring the protein content by Bradford assay (Biorad, Hercules, CA, USA).

### Expression and purification of recombinant enzymes

A soluble epitope tagged from of human TNAP was produced and purified as described previously.(21) Recombinant epitope-tagged human PHOSPHO1 was expressed and purified as described previously.(18) A plasmid containing residues 85 to 905 of mouse NPP1 fused to the rat NPP2 N-terminal signaling peptide (33 residues) was kindly provided by Dr Mathieu Bollen (Leuven, Belgium). To express NPP1, the 2.46 kb cDNA fragment containing the coding sequence of the extracellular region of the rat/mouse NPP1 fusion product was amplified and cloned into the pcDNA3.1/D-V5-His-TOPO expression vector (Invitrogen). The epitope-tagged NPP1 was expressed and secreted from 293 cells and purified from conditioned medium by Ni-column and size-exclusion fast protein liquid chromatography (FPLC).

### Enzymatic assays

*p*-Nitrophenylphosphatase (*p*-NPP) activity was assayed discontinuously at 37°C by following the liberation of *p*-nitrophenolate ion (ɛ 1 M, pH 13 = 17,600 M^*−*1^ cm^*−*1^) at 410 nm. Standard conditions were 50 mmol/L Tris buffer, pH 7.4, containing 2 mmol/L MgCl_2_ and 10 mmol/L *p*-NPP or *p*NP-TMP in a final volume of 0.5 mL. The reaction was initiated by the addition of the enzyme and stopped with 0.5 mL of 1 mol/L NaOH at appropriate time intervals.(22) ATPase, pyrophosphatase (PP_i_ase), ADPase, and AMPase activities were assayed discontinuously by measuring the amount of inorganic phosphate liberated according to a procedure described previously,(23) adjusting the assay medium to a final volume of 0.5 mL. Standard assay conditions were 50 mmol/L Tris buffer, pH 7.4, containing 2 mmol/L MgCl_2_ and substrate. The reaction was initiated by the addition of the enzyme and stopped with 0.25 mL of cold 30% trichloracetic acid (TCA) at appropriate time intervals. The reaction mixture was centrifuged at 4000 × *g*, and phosphate was quantified in the supernatant after pH neutralization with 0.1 mol/L NaOH. The following inhibitors also were used, alone or in combination, to assess the contribution of individual MV enzymes to ATP hydrolysis: 1 mmol/L lanzoprazole (inhibitor of PHOSPHO1),(19) 30 µmol/L MLS-0038949 (inhibitor of TNAP),(24) and 0.25 mmol/L suramin (inhibitor of NPP1).(25) All determinations were carried out in duplicate, and the initial velocities were constant for at least 90 minutes, provided that less than 5% of substrate was hydrolyzed. Controls without added enzyme were included in each experiment to allow for the nonenzymatic hydrolysis of substrate. One enzyme unit (1 U) is defined as the amount of enzyme hydrolyzing 1.0 nmol of substrate per minute at 37°C per milliliter or milligram of protein, as specified in the text. Maximum velocity (*V*_m_), apparent dissociation constant (*K*_0.5_), and Hill coefficient (n) obtained from substrate hydrolysis were calculated as described previously.(26) Data were reported as the mean of triplicate measurements of three different enzyme preparations.

### Measurement of nucleotide hydrolysis by HPLC

The hydrolysis of ATP, ADP, and AMP was analyzed using WT and TNAP-, NPP1-, and PHOSPHO1-deficient MVs. Hydrolysis was determined at 37°C in 50 mmol/L Tris HCl buffer, pH 7.4, containing 2 mmol/L MgCl_2_ and 2 mmol/L ATP or ADP. The reaction was started by addition of the MVs, and at predetermined intervals, samples were removed and assayed immediately. Nucleotides were separated and quantified by HPLC, injecting a 20 µL aliquot of the sample into a C_18_ reversed-phase column (Shimadzu) and eluting it at 1.5 mL/min, with the mobile phase consisting of 50 mmol/L potassium–phosphate buffer (pH 6.4), 5 mmol/L tetrabutylammonium–hydrogen sulfate, and 18 % (v/v) methanol. The absorbance at 260 nm was monitored continuously, and the nucleotide concentrations were determined from the area under the absorbance peaks.

### Calcification assay

The calcification ability of MVs was assayed by a nonradioactive calcium phosphate deposition assay described previously.(27) Briefly, 30 µg of MV proteins was first lysed in deionized water to release the enzymes present inside MVs and then incubated in a calcifying solution containing 2.2 mmol/L Ca^2+^ and 1.6 mmol/L P_i_ for 5.5 hours at 37°C. Incubation was terminated by centrifugation at 8800 × *g* for 30 minutes to coprecipitate MVs and calcium phosphate mineral formed during incubation. The pellet then was solubilized with 0.6 mol/L HCl for 24 hours. The calcium content of the HCl supernatant was determined colorimetrically by the *O*-cresolpthalein complexone method (Calcium Kit, Procedure 0150, Stanbio Laboratories, Boerne, TX, USA).

## Results

**Fig. 1:**
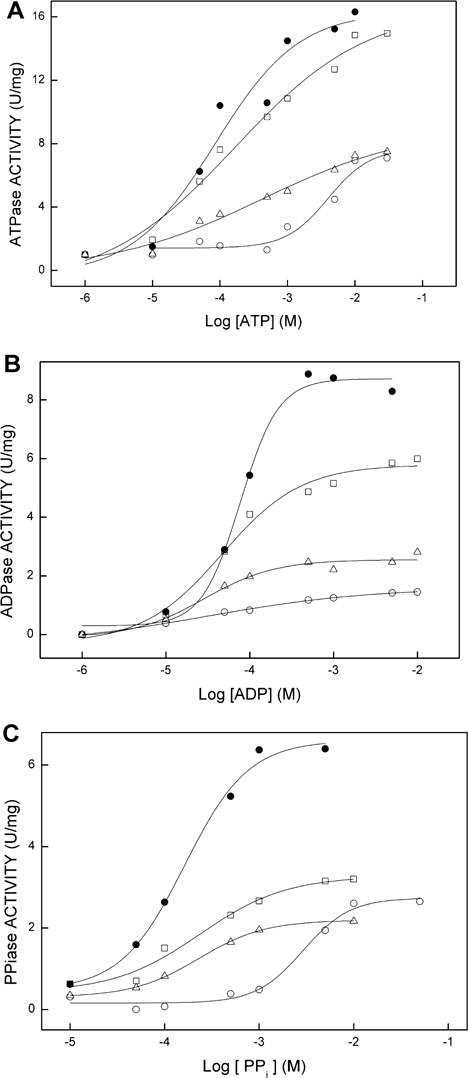
Effect of increasing concentrations of ATP (*A*), ADP (*B*), and PP_i_ (*C*) on the P_i_-generating activity of the MVs: WT (•); TNAP-deficient (○); NPP1-deficient (□), and PHOSPHO1-deficient (Δ). Assays were done at 37°C and buffered with 50 mmol/L Tris HCl, pH 7.4, containing 2 mmol/L MgCl_2_ and substrate and released P_i_ measured as described in “Materials and Methods.”

The effects of increasing concentrations of ATP, ADP, and PP_i_ on the P_i_-generating activity of WT and genetically modified MVs deficient in TNAP, NPP1, or PHOSPHO1 are shown in Fig. 1, and the kinetic parameters are summarized in Table 1. P_i_-generating activity values were highest when ATP was used as a substrate for all MV preparations. At concentrations above 10 mmol/L substrate, we observed inhibition of ATPase activity (not shown). Similar rates of hydrolysis were observed for NPP1-deficient and WT MVs, indicating negligible catalysis of ATP by NPP1 (see 1*A*). In contrast, at 1 mmol/L ATP, no catalysis occurred by *Tnap*^*−*/*−*^ MVs, whereas maximum catalysis was found for WT MVs at this concentration, pointing to a crucial role for TNAP in substrate conversion (see 1*A*). *Phospho1*^*−*/*−*^ MVs showed intermediate ATP catalysis, with a substrate saturation curve extending over a wide range of concentrations. Correspondingly, the *K*_0.5_ for ATP in *Tnap*^*−*/*−*^ MVs was approximately 50-fold higher than that of WT MVs (see Table 1). The lowest *V*_m_ was found for *Tnap*^*−*/*−*^ MVs (see Table 1). We used enzyme inhibitors as a complementary means of validating the contributions of TNAP, NPP1, and PHOSPHO1 to P_i_ generation using ATP as substrate. Compound MLS-0038949 at 30 µmol/L inhibits the ATPase activity of purified TNAP by 89.0%, of purified NPP1 by 62%, and of purified PHOSPHO1 by 15.8% at pH 7.4. Suramin at a concentration of 0.25 mmol/L inhibited the ATPase activity of purified NPP1 by 81.8%, of purified PHOSPHO1 by 71%, and of purified TNAP by 19.7%. Lanzoprazole inhibited the ATPase activity of purified PHOSPHO1 by 52.8%, of purified NPP1 by 14.4%, and of purified TNAP by 2.4%. Hydrolysis of 1 mmol/L ATP by WT MVs was completely prevented by the simultaneous use of 30 µmol/L MLS-0038949, 0.25 mmol/L suramin, and 1 mmol/L lanzoprazole.

**Table 1:** Kinetic Parameters for the Hydrolysis of ATP, ADP, and PP_i_ by Purified TNAP and NPP1 and by WT and Genetically Modified MVs

				Matrix Vesicles (MVs)			
				
Substrates	Kinetic parameters	Purified NPP1	Purified TNAP	*Tnap*^*−*/*−*^	*Npp1*^*−*/*−*^	*Phospho1*^*−*/*−*^	WT
ATP	*V*_m_ (U/mg)	20.9 ± 0.6	33.4 ± 0.9	7.7 ± 0.5	15.2 ± 0.4	9.0 ± 0.3	16.6 ± 0.2
	*K*_0.5_ (mM)	0.603 ± 0.05	0.030 ± 0.008	4.0 ± 0.11	0.21 ± 0.02	0.42 ± 0.03	0.085 ± 0.02
	n	0.688 ± 0.08	1.4 ± 0.05				
ADP	*V*_m_ (U/mg)	30.0 ± 0.8	39.4 ± 0.5	1.6 ± 0.1	5.8 ± 0.2	2.6 ± 0.1	8.7 ± 0.3
	*K*_0.5_ (mM)	0.245 ± 0.4	0.040 ± 0.003	0.031 ± 0.003	0.047 ± 0.005	0.030 ± 0.004	0.077 ± 0.003
	n	1.06 ± 0.05	1.3 ± 0.06				
PP_i_	*V*_m_ (U/mg)	44.4 ± 0.4	72.1 ± 0.9	2.7 ± 0.2	3.3 ± 0.1	2.2 ± 0.2	6.6 ± 0.2
	*K*_0.5_ (mM)	1.14 ± 0.07	0.15 ± 0.4	2.9 ± 0.1	0.24 ± 0.3	0.23 ± 0.3	0.16 ± 0.4
	n	0.859 ± 0.05	0.91 ± 0.03				

*Note*: U/mg corresponds to the nmoles of phosphate released per minute per milligram of total protein. Data are reported as the mean ± SD of triplicate measurements of different matrix vesicle (MV) preparations.

Overall, ADP was a weaker substrate, but we observed similar relative P_i_-generating activities as for ATP for all MVs (see 1*B*). WT MVs were most active, whereas the lowest rate of P_i_ generation was found for *Tnap*^*−*/*−*^ MVs. Inhibition of ADPase activity was evident at ADP concentrations above 5 mmol/L for WT MVs and above 50 mmol/L for the other MVs (not shown). Both the *Tnap*^*−*/*−*^ and *Phospho1*^*−*/*−*^ MVs were poorly active but with comparable *K*_0.5_ values (see Table 1).

With PP_i_ as the substrate, P_i_ generation was significantly reduced for all genetically modified MVs compared with WT MVs. The *K*_0.5_ value obtained for *Tnap*^*−*/*−*^ MVs was about 20-fold higher than for WT MVs, and *Npp1*^*−*/*−*^ and *Phospho1*^*−*/*−*^ MVs displayed similar affinity constants (see 1*C* and Table 1). We observed inhibition of PP_i_ase activity at PP_i_ concentrations above 5 mmol/L for WT MVs, above 10 mmol/L for *Phospho1*^*−*/*−*^ MVs, and above 50 mmol/L for *Tnap*^*−*/*−*^ and *Npp1*^*−*/*−*^ MVs.

**Fig. 2:**
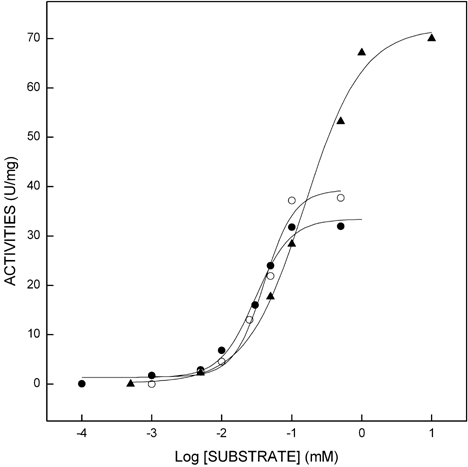
Effect of increasing concentrations of ATP (•), ADP (○), and PP_i_ (▴) on the P_i_-generating activity of purified TNAP. Assays were done at 37°C and buffered with 50 mmol/L Tris HCl, pH 7.4, containing 2 mmol/L MgCl_2_ and substrate and P_i_ measured as described in “Materials and Methods.” Note that for PP_i_, 2 mol of P_i_ is generated per mole of substrate.

**Fig. 3:**
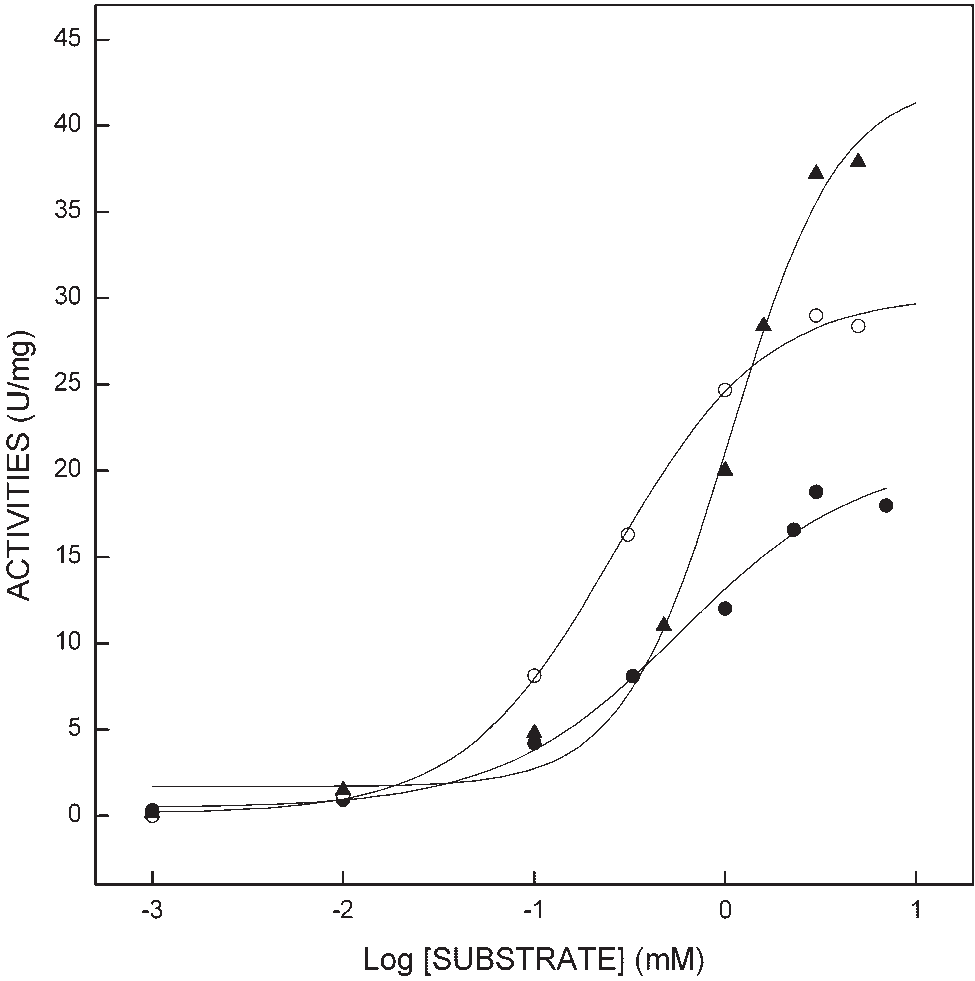
Effect of increasing concentrations of ATP (•), ADP (○), and PP_i_ (▴) on the P_i_-generating activity of purified NPP1. Assays were done at 37°C and buffered with 50 mmol/L Tris HCl, pH 7.4, containing 2 mmol/L MgCl_2_ and substrate and P_i_ measured as described in “Materials and Methods.” Note that for PP_i_, 2 mol of P_i_ is generated per mole of substrate.

To investigate the strong loss of hydrolytic activity in *Tnap*^*−*/*−*^ and *Phospho1*^*−*/*−*^ MVs in more detail, the kinetic properties of purified TNAP, NPP1, and PHOSPHO1 were analyzed under the same kinetic conditions. The saturation curves for P_i_ generation owing to hydrolysis of ATP, ADP, and PP_i_ by isolated TNAP (Fig. 2) show highly efficient catalysis for all three substrates, with *K*_0.5_ values (see Table 1) close to the values measured in WT MVs and positive cooperativity for ATP and ADP (see Table 1). We found that NPP1 is able to hydrolyze ATP, ADP, and PP_i_ with high velocity but lower affinity than TNAP (see Table 1), and negative cooperativity was observed for the hydrolysis of ATP and PP_i_ (Fig. 3). In contrast, compared with the catalytic efficiency of purified TNAP, purified PHOSPHO1 behaved poorly, with *V*_m_ values of 0.57, 0.42, and 0.31 U/mg for the hydrolysis of ATP, ADP, and PP_i_, respectively (not shown). These measurements confirmed that PHOSPHO1 metabolizes ATP very inefficiently. Yet the data shown earlier (see Table 1 and 1*A*) indicated reduced utilization of ATP by *Phospho1*^*−*/*−*^ MVs. To understand this change, we measured the levels of TNAP activity for WT and *Phospho1*^*−/−*^ MV preparations (4*A*). We observed a reduction of up to 70% in TNAP activity in *Phospho1*^*−*/*−*^ MVs. Calcification assays indicated a reduction in matrix calcification by both *Tnap*^*−*/*−*^ and *Phospho1*^*−*/*−*^ MVs, whereas *Npp1*^*−*/*−*^ MVs showed increased calcification compared with WT MVs (see 4*B*).

**Fig. 4:**
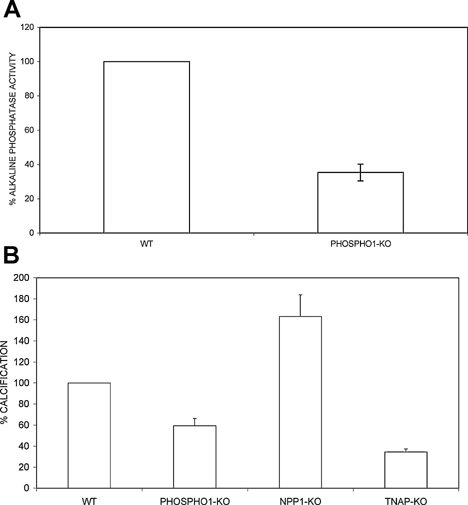
(*A*) Alkaline phosphatase activity in WT and PHOSPHO1-deficient MVs. Assays were buffered with 1 mol/L DEA, pH 9.6, containing 1 mmol/L MgCl_2_, 20 µmol/L ZnCl_2_, and 10 mmol/L *p*-NPP. Liberated *p*-nitrophenolate was measured at 410 nm as described in “Materials and Methods.” (*B*) Calcification ability by WT, PHOSPHO1-deficient, NPP1-deficient, and TNAP-deficient MVs, assayed as described in “Materials and Methods.”

**Fig. 5:**
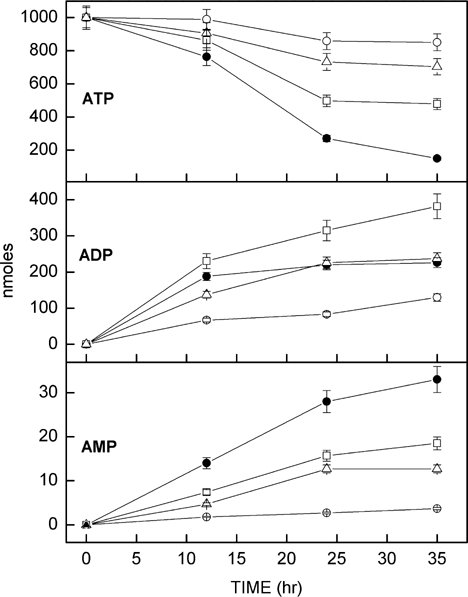
Amounts of hydrolyzed ATP and produced ADP and AMP by MVs: WT (•), TNAP-deficient (○), NPP1-deficient (□), and PHOSPHO1-deficient (Δ), monitored by HPLC analysis as described in “Materials and Methods.”

**Fig. 6:**
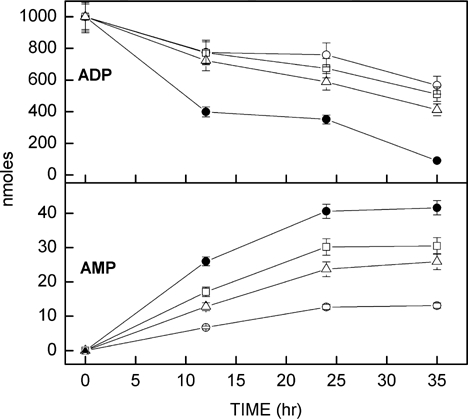
Amounts of hydrolyzed ADP and produced AMP for MVs: WT (•), TNAP-deficient (○), NPP1-deficient (□), and PHOSPHO1-deficient (Δ), monitored by HPLC analysis as described in “Materials and Methods.”

To investigate in more detail how the simultaneous presence of various phosphatases in MVs would dictate nucleotide hydrolysis, we monitored the formation of reaction intermediates as a function of time during hydrolysis of ATP and ADP via separation and quantification of products by HPLC. The amounts of hydrolyzed ATP or ADP and the amounts of ADP and/or AMP produced for the different MV preparations are shown for the hydrolysis of ATP (Fig. 5) and ADP (Fig. 6). HPLC analysis demonstrated that ATP is hydrolyzed most efficiently (approximately 80% of the initial amount) by WT MVs, in agreement with the data shown in Fig. 1. *Tnap*^*−*/*−*^ MVs were least active (utilization of substrate approximately 20%). In agreement with our findings in Fig. 1, *Phospho1*^*−*/*−*^ MVs and *Tnap*^*−*/*−*^ MVs were similarly inactive. The amount of ADP produced was similar for *Phospho1*^*−*/*−*^ and WT MVs and was lowest for the *Tnap*^*−*/*−*^ MVs (see Fig. 5, ADP). AMP was produced to very low levels compared with the amount of ADP produced and, although different for all MVs studied, was most elevated for the WT MVs (see Fig. 5, AMP). AMP accumulated only to low levels (40 nmol) because it was hydrolyzed itself with high catalytic efficiency to adenosine and phosphate. Direct analytic phosphate measurements during AMP hydrolysis showed a rise in P_i_ paralleling the drop in the concentration of AMP with time (not shown). The amounts of P_i_ released after 36 hours of ATP hydrolysis were 987 ± 49, 177 ± 9, 423 ± 21, and 362 ± 18 nmol for WT, *Tnap*^*−*/*−*^, *Npp1*^*−*/*−*^, and *Phospho1*^*−*/*−*^ MVs, respectively. When ADP was used as a substrate, the highest rate of catalysis was observed (see Fig. 6, ADP), again for WT MVs (approximately 90% of the initial amount), whereas the rate was comparatively lower (approximately 40%) for all genetically modified MVs. The amount of AMP produced was significantly different for all MVs and practically insignificant for *Tnap*^*−/−*^ MVs (see Fig. 6, AMP). Quantification of released P_i_ after 36 hours of reaction revealed 610 ± 31, 160 ± 8, 695 ± 34, and 229 ± 11 nmol for WT, *Tnap*^*−*/*−*^, *Npp1*^*−*/*−*^, and *Phospho1*^*−*/*−*^ MVs, respectively. Comparison of the rate of AMP formation during catalysis of ATP (see Fig. 5) and ADP (see Fig. 6) shows that these are comparable, in agreement with our finding that the rate-limiting step in our setup is not the hydrolysis of ATP to ADP (see 1*A, B*). Since, in addition, the ADP-to-AMP conversion and hydrolysis of AMP generate P_i_, the rate of P_i_ formation is best described by the generation of ADP from ATP (see 5*B*) and by the disappearance rate of ADP (see 6*A*). Hence the higher P_i_ accumulation for ATP hydrolysis, the higher are the number of phosphate molecules in ATP over ADP.

## Discussion

As we strive to understand the detailed roles of TNAP, NPP1, PHOSPHO1, and other important MV enzymes in the initiation of biomineralization, we must keep in mind the microenvironment in which these enzymes function, which can have a profound effect on their biologic properties. Recent data showed that the location of TNAP on the membrane of MVs plays a role in determining substrate selectivity in this microcompartment.(28) Those data indicated that assays of TNAP bound to MVs or to liposome-based systems may be more biologically relevant than assays done on solubilized enzyme preparations. Furthermore, under physiologic conditions, these enzymes function in concert when confronted with a physiologic mixture of substrates, and it would be relevant to understand their relative efficiency for individual substrates when present together in their native biologic compartment. Therefore, in this study we have compared the kinetic behavior of native WT MVs with that of MVs deficient in TNAP, NPP1, or PHOSPHO1 when confronted with physiologic substrates, that is, ATP, ADP, and PP_i_, at physiologic pH.

Comparison of the catalytic activities measured for all deficient and WT MVs identified ATP as the main MV substrate, in agreement with earlier observations.(29) We observed previously that ATP-dependent ^45^Ca precipitation was decreased in calvarial osteoblast-derived MVs from TNAP-deficient mice,(9) an observation that we attributed to the increases in MV PP_i_ content resulting from reduced PP_i_ hydrolysis in *Tnap*^*−*/*−*^ MVs. However, our present study clearly indicates that TNAP is not only an efficient PP_i_ase but that at the level of MVs it also functions as a potent ATPase and ADPase. We also observed reduced utilization of ATP, resulting in decreased calcification, by *Phospho1*^*−*/*−*^ MVs. This experimental result is explained not by decreased PHOSPHO1-mediated hydrolysis of ATP but rather by the up to 70% reduction in TNAP activity that can be found in *Phospho1*^*−*/*−*^ osteoblasts and their MVs. *Npp1*^*−/−*^ MVs show the typical hypercalcification behavior caused by their reduced content of PP_i_.(10)

Analysis of the Hill coefficients for the catalysis of ATP by the various MVs was suggestive of a slight negative cooperativity (n ranging from 0.44 to 1), which was not investigated further in the present study. The *K*_0.5_ values obtained for the hydrolysis of ATP by enzymes present in WT MVs were very similar to those reported for purified phosphatidylinositol phospholipase C-released alkaline phosphatase from membrane from rat osseous Plate(23) and those obtained for high-affinity substrate site from membrane-bound enzyme obtained from osseous plate.(29) The *K*_0.5_ value obtained from TNAP-deficient MVs instead was very similar to that of the low-affinity substrate site reported for ATPase activity of rat osseous plate.(30) Several lines of evidence had suggested that specific ATPase and TNAP from MVs were different enzymes.(31) While hydrolysis of ATP was reduced in the absence of TNAP and PHOSPHO1, significant residual ATPase activity remains, suggesting that another ATPase is still present in the MV membrane, either another specific ATPase(31) or NPP1.(32) In an attempt to clarify this issue further, we resorted to using inhibitors of the three phosphatases studied in this article. While the use of inhibitors is commonplace, this approach may lead to confounding results owing to potential off-target effects. Compound MLS-0038949 was selected through a comprehensive high-throughput screening campaign to identify specific inhibitors of TNAP.(24) This compound was found to be selective for TNAP and not to affect other targets, except CYP2C19, a member of the cytochrome P450 mixed-function oxidase system,(24,33) and we show here that it also inhibits NPP1 and PHOSPHO1 function to some extent. Suramin has been described as a specific NPP1 inhibitor, and we report here that it also inhibits TNAP and PHOSPHO1, besides being a nonselective P_2_ receptor antagonist.(34) Lanzoprazole was identified during a screening for small-molecule inhibitors as being specific for PHOSPHO1 and not affecting TNAP activity,(18) but as we have determined in the present study, it partially inhibits NPP1 activity. In addition, lansoprazole belongs to the family of 2-(2-pyridylmethylsulfinyl)-1*H*-benzimidazoles and is a well-known inhibitor of H^+^,K^+^-ATPase of stomach parietal cells.(35) While all these inhibitors can have off-target effects in vivo and documented cross-specificities for related phosphatases, their restricted and judicious use to further dissect the role of phosphatases involved in MV-mediated calcification appears justified. Thus, while it remains possible that these compounds may be inhibiting some yet unknown MV phosphatase, the complete inhibition of ATPase activity in WT MVs by the simultaneous use of MLS-0038949, suramin, and lanzoprazole appears to indicate that all ATPase activity in WT MVs can be accounted for by these three MV phosphatases, that is, TNAP, NPP1, and PHOSPHO1.

The high P_i_-generating activity of NPP1 when confronted with ATP, ADP, or PP_i_ indeed supports the contention that NPP1 can act as an ATPase, ADPase, and even PP_i_ase on the surface of MVs (see Table 1) functioning as a backup metabolic pathway when TNAP activity is suboptimal. Indeed, the similar hydrolysis rates observed for *Npp1*^*−/−*^ and WT MVs suggests that ATP is hydrolyzed more efficiently by TNAP than by NPP1, as demonstrated by the *K*_0.5_ values for these genetically deficient MVs (see Table 1). The same response was observed when using ADP as substrate, indicating that TNAP is also the main enzyme hydrolyzing this substrate (see 1*B*). This interpretation is further supported by the 20-fold (ATP) and 6-fold (ADP) higher *K*_0.5_ values measured for purified NPP1 versus TNAP (see Table 1), which confer the latter enzyme with a competitive advantage for these nucleotide substrates. The *K*_0.5_ and Michaelis-Menten behavior for hydrolysis of PP_i_ by TNAP in WT MVs are very similar to those obtained for PP_i_ase activity from rat osseous plate alkaline phosphatase in the presence of 0.1 mmol/L magnesium ions,(36,37) indicating that TNAP can indeed hydrolyze PP_i_ at physiologic conditions. The kinetic parameters measured for the TNAP-deficient MVs suggest that the remaining hydrolysis of PP_i_ is accounted for by the PP_i_ase activity of NPP1 in these vesicles. The ATPase and PP_i_ase roles of NPP1 (producing P_i_, not PP_i_) are visible only in the absence of TNAP (see Fig. 1 and Table 1). Thus the presence of such residual compensatory ATPase and PP_i_ase activities very likely explains why TNAP-deficient mice are born with a mineralized skeleton and only begin to develop the classic features of hypophosphatasia on postnatal days 6 to 8.(14,15)

**Fig. 7:**
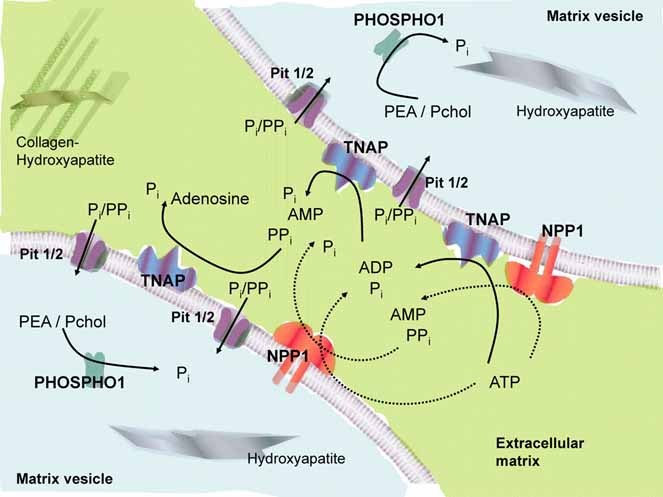
Schematic representation of the most favored enzymatic reactions in the MV compartment at physiologic pH. We surmise that HA seed crystals are formed in the sheltered interior of MVs favored by the P_i_-generating ability of PHOSPHO1, as well as by the function of Pit1/2 phosphate transporters. While NPP1 clearly has a role in PP_i_-generation at the level of the chondrocyte and osteoblast membranes, at the level of MVs NPP1 does not use ATP efficiently. Instead, it is TNAP that has a major role both as a PP_i_ase and an ATPase/ADPase and thus participates in the calcification process both by restricting the concentration of extracellular PP_i_ and by also contributing to the P_i_ pool available for calcification. Thick lines indicate the most favored reactions, whereas dotted lines indicate alternative enzymatic pathways, such as the ability of NPP1 to act as a phosphatase.

The hydrolysis of ATP, ADP, and PP_i_ at pH 7.4 is of physiologic significance for the calcification process because it is known that TNAP, NPP1, and PHOSPHO1 play crucial roles in normal bone mineralization. This is well exemplified in this study by the reduced calcification ability of TNAP- and PHOSPHO1-deficient MVs, whereas NPP1-deficient MVs hypercalcify (see 4*B*). Our working model is that bone mineralization is first initiated within the lumen of MVs. In a second step, HA crystals grow beyond the confines of the MVs and become exposed to the extracellular milieu, where they continue to propagate along collagen fibrils (Fig. 7). Hydroxyapatite seed crystals are formed in the sheltered interior of MVs favored by the P_i_-generating activity of PHOSPHO1, as well as by the transport function of Pit 1/2 transporters. While NPP1 clearly has a role in PP_i_ generation at the level of the chondrocyte and osteoblast membranes, at the level of MVs, NPP1 does not appear to use ATP efficiently. Instead, it is TNAP that has a major role both as a pyrophosphatase and as an ATPase and ADPase and thus participates in the calcification process both by restricting the concentration of extracellular PP_i_ and by simultaneously contributing to the P_i_ pool available for calcification via its enzymatic action on ATP, ADP, and PP_i_. The cooperativity as well as competition of TNAP, NPP1, and PHOSPHO1 for these biomineralization substrates provides an additional level of regulation of metabolite flow for control of the calcification process.

## References

[1] Glimcher MJ (2006). Bone: nature of the calcium phosphate crystals and cellular, structural, and physical chemical mechanisms in their formation. Rev Miner Geochem..

[2] Anderson HC, Garimella R, Tague SE (2005). The role of matrix vesicles in growth plate development and biomineralization. Front Biosci..

[3] Schinke T, McKee MD, Karsenty G (1999). Extracellular matrix calcification: where is the action?. Nat Genet..

[4] Giachelli CM (2005). Inducers and inhibitors of biomineralization: lessons from pathological calcification. Orthod Craniofac Res..

[5] Meyer JL (1984). Studies on matrix vesicles isolated from chick epiphyseal cartilage: association of pyrophosphatase and ATPase activities with alkaline phosphatase. Arch Biochem Biophys..

[6] Terkeltaub RA (2001). Inorganic pyrophosphate generation and disposition in pathophysiology. Am J Physiol Cell Physiol..

[7] Ho AM, Johnson MD, Kingsley DM (2000). Role of the mouse ank gene in control of tissue calcification and arthritis. Science..

[8] Millán JL (2006). Mammalian Alkaline Phosphatases: From Biology to applications in Medicine and Biotechnology.

[9] Johnson KA, Hessle L, Vaingankar S, Wennberg C, Mauro S, Narisawa S, Goding JW, Sano K, Millán JL, Terkeltaub R (2000). Osteoblast tissue-nonspecific alkaline phosphatase (TNAP) antagonizes and regulates PC-1. Am J Phys Reg Integr Comp Phys..

[10] Hessle L, Johnson KA, Anderson HC, Narisawa S, Sali A, Goding JW, Terkeltaub R, Millán JL (2002). Tissue-nonspecific alkaline phosphatase and plasma cell membrane glycoprotein-1 are central antagonistic regulators of bone mineralization. Proc Natl Acad Sci USA..

[11] Murshed M, Harmey D, Millán JL, McKee MD, Karsenty G (2005). Unique coexpression in osteoblasts of broadly expressed genes accounts for the spatial restriction of ECM mineralization to bone. Genes Dev..

[12] Harmey D, Hessle L, Narisawa S, Johnson KA, Terkeltaub R, Millán JL (2004). Concerted regulation of inorganic pyrophosphate and osteopontin by *Akp2*, *Enpp1* and *Ank*: an integrated model of the pathogenesis of mineralization disorders. Am J Pathol..

[13] Narisawa S, Harmey D, Yadav MC, O'Neill WC, Hoylaerts MF, Millán JL (2007). Novel inhibitors of alkaline phosphatase suppress vascular smooth muscle cell calcification. J Bone Miner Res..

[14] Narisawa S, Fröhlander N, Millán JL (1997). Inactivation of two mouse alkaline phosphatase genes and establishment of a mouse model of infantile hypophosphatasia. Dev Dyn..

[15] Fedde KN, Blair L, Silverstein J, Colburn SP, Ryan LM, Weinstein RS, Waymire K, Narisawa S, Millán JL, MacGregor GR, Whyte MP (1999). Alkaline phosphatase knock-out mice recapitulate the metabolic and skeletal defects of infantile hypophosphatasia. J Bone Miner Res..

[16] Anderson HC, Sipe JE, Hessle L, Dhamayamraju R, Atti E, Camacho NP, Millán JL (2004). Impaired calcification around matrix vesicles of growth plate and bone in alkaline phosphatase-deficient mice. Am J Pathol..

[17] Anderson HC, Harmey D, Camacho NP, Garimella R, Sipe JB, Tague S, Bi X, Johnson K, Terkeltaub R, Millán JL (2005). Sustained Osteomalacia of Long Bones Despite Major Improvement in Other Hypophosphatasia-Related Mineral Deficits in TNAP/NPP1 Double-Deficient Mice. Am J Pathol..

[18] Roberts SJ, Stewart AJ, Sadler PJ, Farquharson C (2004). Human PHOSPHO1 displays high specific phosphoethanolamine and phosphocholine phosphatase acitivity. Biochem J..

[19] Roberts S, Narisawa S, Harmey D, Millán JL, Farquharson C (2007). Functional involvement of PHOSPHO1 in matrix vesicle-mediated skeletal mineralization. J Bone Miner Res..

[20] Sali A, Favaloro JM, Terkeltaub R, Goding JW, Vanduffel L, Lemmems R (1999). Germline deletion of the nucleoside triphosphate (NTPPPH) plasma cell membrane glycoprotein-1 (PC-1) produces abnormal calcification of periarticular tissues. Ecto-ATPases and Related Ectoenzymes.

[21] Di Mauro S, Manes T, Hessle H, Kozlenkov A, Pizauro JM, Hoylaerts MF, Millán JL (2002). Kinetic characterization of hypophosphatasia mutations with physiological substrates. J Bone Min Res..

[22] Camolezi FL, Daghastanli KRP, Magalhães PP, Pizauro JM, Ciancaglini P (2002). Construction of an alkaline phosphatase-liposome system: a tool for biomineralization study. Int J Biochem Cell Biol..

[23] Pizauro JM, Ciancaglini P, Leone FA (1995). Characterization of the phosphatidylinositol-specific phospholipase C-released form of rat osseous plate alkaline phosphatase and its possible significance on endochondral ossification. Mol Cell Biochem..

[24] Sergienko E, Su Y, Chan X, Brown B, Hurder A, Narisawa S, Millán JL (2009). Identification and characterization of novel tissue-nonspecific alkaline phosphatase inhibitors with diverse modes of action. J Biomol Screen..

[25] Rücker B, Almeida ME, Libermann TA, Zerbini LF, Wink MR, Sarkis JJ (2007). Biochemical characterization of ecto-nucleotide pyrophosphatase/phosphodiesterase (E-NPP, E.C. 3.1.4.1) from rat heart left ventricle. Mol Cell Biochem..

[26] Leone FA, Baranauskas JA, Furriel RPM, Borin IA (2005). SigrafW: An easy-to-use program for fitting enzyme kinetic data. Biochem Mol Educ..

[27] Garimella R, Sipe JB, Anderson HC (2004). A simple and non-radioactive technique to study the effect of monophosphoesters on matrix vesicle-mediated calcification. Biol Proced..

[28] Ciancaglini P, Simão AMS, Camolezi FL, Millán JL, Pizauro JM (2006). Contribution of matrix vesicles and alkaline phosphatase to ectopic bone formation. Brazilian J Med Biol Res..

[29] Hsu HH, Anderson HC (1996). Evidence of the presence of a specific ATPase responsible for ATP-initiated calcification by matrix vesicles isolated from cartilage and bone. J Biol Chem..

[30] Pizauro JM, Ciancaglini P, Leone FA (1993). Allosteric modulation by ATP, calcium and magnesium ions of rat osseous plate alkaline phosphatase. Biochim Biophys Acta..

[31] Pizauro JM, Demenis MA, Ciancaglini P, Leone FA (1998). Kinetic characterization of a membrane specific ATPase from rat osseous plate and its possible significance on endochodral ossification. Biochim Biophys Acta..

[32] Gijsbers R, Ceulemans H, Stalmans W, Bollen M (2001). Structural and catalytic similarities between nucleotide pyrophosphatase/phosphodiesterase and alkaline phosphatases. J Biol Chem..

[33] Dahl R, Sergienko EA, Mostofi YS, Yang L, Su Y, Simão AM, Narisawa S, Brown B, Mangravita-Novo A, Smith LH, O'Neill WC, Millán JL, Cosford NDP (2009). Discovery and Validation of a Series of Aryl Sulfonamides as Selective Inhibitors of Tissue-Nonspecific Alkaline Phosphatase (TNAP). J Med Chem..

[34] Grobben B, Claes P, Roymans D, Esmans EL, Van Onckelen H, Slegers H (2000). Ecto-nucleotide pyrophosphatase modulates the purinoceptor-mediated signal transduction and is inhibited by purinoceptor antagonists. Br J Pharm..

[35] Nagaya H, Satoh H, Maki Y (1990). Possible mechanism for the inhibition of acid formation by the proton pump inhibitor AG-1749 in isolated canine parietal cells. J Pharmacol Exp Ther..

[36] Rezende LA, Ciancaglini P, Pizauro JM, Leone FA (1998). Inorganic pyrophosphate-phosphohydrolytic activity associated with rat osseous plate alkaline phosphatase. Cell Mol Biol (Noisy-le-Grand)..

[37] Leone FA, Rezende LA, Ciancaglini P, Pizauro JM (1998). Allosteric modulation of pyrophosphatase activity of rat osseous plate alkaline phosphatase by magnesium ions. Int J Biochem Cell Biol..

